# Fluorescence imaging of hepatocellular carcinoma with a specific probe of COX-2†

**DOI:** 10.1039/c7ra07819f

**Published:** 2018-01-03

**Authors:** Haibo Wang, Chengyong Dong, Keqiu Jiang, Shuangzhe Zhang, Fei Long, Rixin Zhang, Deguang Sun, Rui Liang, Zhenming Gao, Shujuan Shao, Liming Wang

**Affiliations:** Division of Hepatobiliary and Pancreatic Surgery, Department of General Surgery, The Second Affiliated Hospital of Dalian Medical University Dalian Liaoning China wangbcc259@163.com +86-411-84671291 +86-411-84671291; Key Laboratory of Fine Chemicals in Dalian University of Technology Dalian Liaoning China; School of Chemistry and Chemical Engineering, Henan Normal University Zhengzhou Henan China; Key Laboratory of Proteomics, Dalian Medical University Dalian Liaoning China

## Abstract

Hepatocellular carcinoma (HCC) is the major subtype of primary liver cancer. Although the standard treatment method based on surgery has generally extended life, it still causes the second and sixth most prevalent cancer-related death in men and women, respectively. The recurrence of cancer caused by unclear resection margins and any remaining undiscovered metastatic nodules should take a large proportion of responsibility for the poor prognosis after resective surgery. Therefore, a practical and effective method that can be used during hepatectomy to specifically identify HCC is a potentially significant area deserving attention. Tests involving fluorescence have been used in many biological systems. In this study, we use a probe that can combine with cyclooxygenase-2 (COX-2) and subsequently emit fluorescence to identify HCC cells and heteroplastic tumors in a mouse model. The results show that this specific probe can clearly differentiate HCC, with differences that could be observed with the naked eye in human samples. The biotechnology of knocking down COX-2 and its inhibitor were used on human HCC cell line SMMC7721, and the outcomes confirmed the above results. The toxic effect also showed that the probe had no harmful effect on normal liver cells. Taken together, our study demonstrates that a COX-2-specific fluorescence probe may be a new and effective method to identify HCC, especially during surgery.

## Abstract 2

Hepatocellular carcinoma (HCC) is the major subtype of primary liver cancer.

## Graphical abstract

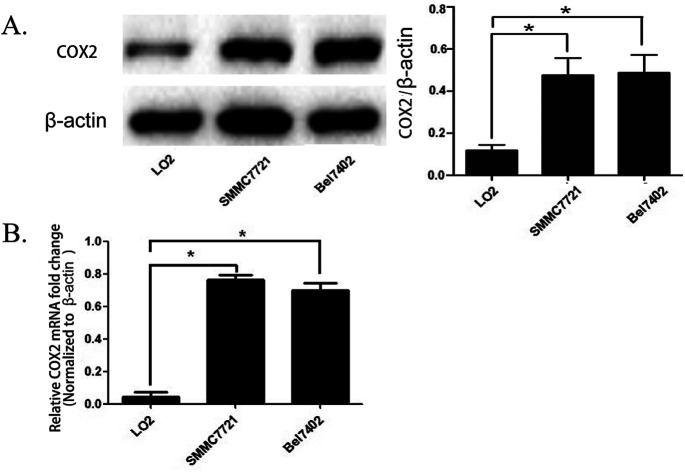

## Introduction

Hepatocellular carcinoma is one of the most common malignancies worldwide. There are more than 700 000 new cases of this type of cancer discovered each year; the 5-year survival rate is less than 20%.^1,2^ One important reason for the poor prognosis is the difficulty in obtaining a positive surgical margin during resection. Although many technologies such as positron emission tomography-computed tomography and histopathological examination of biopsies have been clinically used, there is still great difficulty in clearly distinguishing the tumor during surgery with the naked eye.^3,4^ It is also difficult to discover a microscopic and unimpressive metastasis, especially when it occurs at a region where resection will not occur.^5^ This highlights the importance of discovering a new method that can be used during surgery to easily delineate the location of tumors and make it easier to find small metastasis.

Fluorescent probes are molecules that can absorb a specific wavelength of light and emit light of a longer wavelength.^6,7^ The use of fluorescent probes in biological research has increased, and the technique is now found in many applications.^8–10^ Because of their high-accuracy and high-selectivity, and especially, non-radioactivity, fluorescent probes may offer a more useful strategy for the diagnosis of tumors.^11,12^

Cyclooxygenase-2 (COX-2) is an induced type of cyclooxygenase. For the most part, this type of enzyme is not expressed in normal tissue, but when stimulated by a tumor or infection, the expression of COX-2 will obviously increase.^13^ Many studies have shown that in tumors from the stomach, colon, and breast, the expression of COX-2 is elevated.^14^ There are also studies that found that COX-2 participates in the processes of oncogenesis, proliferation, and migration in hepatocellular carcinoma, and the quantity of COX-2 is increased during the initial, middle, and advanced phases.^15,16^ Therefore, COX-2 may be an appropriate candidate to target in the fluorescence imaging of hepatocellular carcinoma.

In our study, we used a COX-2-specific probe (COX-2 FP) that was compounded by the State Key Laboratory of Fine Chemicals in Dalian University of Technology. In the probe, the fluorophore is connected to COX-2 as a target molecule and acts as a marker for analysis with fluorescence microscopy. When the probe connects to COX-2 in a sample, its structure changes and the exposed fluorophore is then able to emit fluorescence.^17^

## Materials and methods

### Fluorescence probe

The COX-2-specific fluorescence probe was compounded by the State Key Laboratory of Fine Chemicals at Dalian University of Technology. The fluorescence molecule was synthesized according to the method of our previous patent (WO2013131235A1).

### Cells and animals

Human hepatocellular carcinoma cell line SMMC7721 was purchased from the American Type Culture Collection (ATCC, Rockville, MD, USA). BEL7402 and human normal liver cells LO2 were obtained from KeyGen Biotech Co. Ltd (Nanjing, China). All cell lines were conserved at the State Key Laboratory Protein Group in Dalian Medical University and were cultured in Dulbecco's modified Eagle's medium (DMEM) supplemented with 10% fetal bovine serum (FBS) and penicillin–streptomycin in a 37 °C, 5% CO2 environment. The cell lines were continuously observed and were collected at the exponential phase for the experiments.

Ten SPFF stage female BALB/c nude mice, with an average age of 6 weeks and weight between 18–22 g, were purchased from the Shanghai Animal Study Institution of the Chinese Academy of Science. The mice were housed in laminar flow cabinets under pathogen-free conditions.

### siRNA knockdown of COX-2 in SMMC7721 cells

COX-2 siRNA with the following sequences for COX-2 siRNA 1 (S1)—sense: 5′-GCU GGG AAG CCU UCU CUA AdTdT-3′ and antisense: 3′-dTd TCG ACC CUU CGG AAG AGA UU-5′; COX-2 siRNA 2 (S2)—sense: 5′-AAC UGC UCA ACA CCC GAA Utt-3′ and antisense: 5′-AUU CCG GUG UUG AGG AGU Utt-3 were purchased from the Guangzhou Jin Wei Biotech Company. Control siRNA in experiments refers to a All-Star non-silencing siRNA (forward sequence: GGGUAUCGACGAUUACAAAUU, reverse sequence: UUUGUAAUCGUCGAUACCCUG) synthesized by Shanghai GenePharma Co. (Shanghai China). Lipofectamine 2000 transfection reagent (Invitrogen, Carlsbad, CA, USA) was used for the transfection of siRNA according to the manufacturer's instruction.

### Xenograft tumor mouse model

The human hepatocellular carcinoma cell line SMMC7721 was used in the xenograft tumor study. The tumor implants were established by subcutaneous injection of 2 × 10^6^ cells suspended in 200 μL of PBS in the nude mice. The mice were observed until small nodules appeared at the injection site. Then, the diameters were measured every three days. The tumor volume was calculated by the formula *V* = 1/2*ab*(*a* + *b*) (*a* = longest diameter and *b* = shortest diameter), and the fluorescence experiment was initiated twenty days after tumor implantation. Additionally, in the supplementary study, the fluorescence experiment was initiated when the tumor diameters reached 4 mm × 4 mm.

All animal maintenance and procedures were carried out in strict accordance with the recommendations established by the Animal Care and Ethics Committee of Dalian Medical University as well as the guidelines of the U.S. National Institutes of Health Guide for the Care and Use of Laboratory Animals. The protocol was approved by the Animal Care and Ethics Committee of Dalian Medical University.

### Fluorescence imaging in cell lines

SMMC7721, BEL7402, and LO2 cells were at the exponential phase for the fluorescence experiments. The cells were collected and seeded into a glass-bottomed dish (Mat Tek, 35 mm dish with 20 mm wells) and cultured for 24 hours. Then, the medium was changed and replaced with medium containing COX-2 FP (5.0 μM), and the dishes were incubated for 30 minutes in a 37 °C, 5% CO2 environment. A fluorescence microscope was used to continuously observe the cells (*λ*_ex_ = 488 nm, *λ*_em_ 550 ± 20 nm).

### Fluorescence imaging in the mouse model

The mice with xenograft tumors were injected with COX-2 FP 50 μL by the tail vein. The probe was dissolved in PBS, and the final concentration was 30 μM. The mice were anesthetized by inhalation of ether 30 minutes after the injection. A small animal *in vivo* imaging system (Night OWL II LB983) was used to collect the fluorescence images with an excitation laser at 480 nm and an emission filter at 550 ± 10 nm. After the image was obtained, the mice were euthanized by cervical dislocation. The xenograft tumor and parts of the liver were obtained and imaged at the same time.

### Fluorescence imaging of human samples

The hepatocellular carcinoma samples were obtained from patients undergoing surgical resection. The normal hepatocellular carcinoma samples came from resection that was performed because of injury. Diagnosis of all samples was performed by pathology. We sprayed COX-2 FP (50 μM) on the samples, waited approximately 5 minutes, and then used a hand-held ultraviolet lamp emitting 365 nm excitation light to observe the phenomenon with the naked eye.

The present study was approved by the Ethics Committees of the Second Affiliated Hospital of Dalian Medical University in accordance with the ethical guidelines of the Declaration of Helsinki. Informed consent was obtained from all human participants of this study.

### Statistical analysis

Statistical calculations were carried out with Prism 6 software (Graph Pad Software Inc.). The mean ± SD was used to show the datum in the figure legends as indicated. The statistical significance of the data is presented as *P* values (*P* < 0.05, namely, *). *P* ≤ 0.05 was considered to indicate a statistically significant difference.

## Results and discussion

### The expression of COX-2 in the human hepatocellular carcinoma cell lines SMMC7721 and BEL7402 is enhanced compared with normal liver cells LO2

**Fig. 1:**
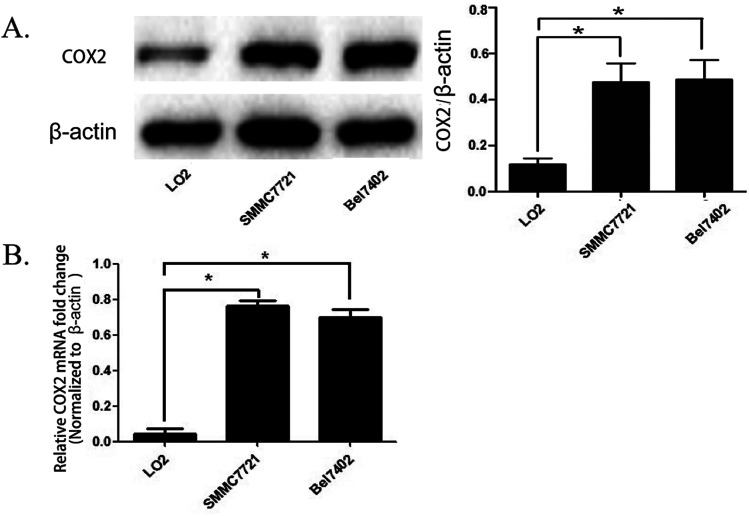
The expression of COX-2 in human hepatocellular carcinoma cell lines SMMC7721 and BEL7402 and normal liver cells LO2. (A) The expression of COX-2 in SMMC7721, BEL7402, and LO2 cells was detected by western blot assay (left panel). The expression of COX-2 was quantified by analysis of the densitometry value with Image Lab 5.0 software and was normalized to β-actin (right panel). The data are presented as the mean ± SD of three separate experiments (*P < 0.05). (B) The expression levels of the mRNAs encoding COX-2 in SMMC7721, BEL7402, and LO2 cells, as determined by real time RT-PCR. Actin mRNA was used to normalize the variability in template loading. The data are reported as the mean ± SD.

We cultured the human hepatocellular carcinoma cell lines SMMC7721, BEL7402, and normal human liver cells LO2. The total mRNA and proteins were obtained when the cell lines were in logarithmic phase and were used for real time PCR and western blot analysis. The results show that the quantity of COX-2 was enhanced in SMMC7721 and BEL7402 cells compared to LO2 cells both at the mRNA and protein level (Fig. 1A and B). The results were the same as that of a previous study showing that the expression of COX-2 is enhanced in most of the hepatocellular carcinoma tissue but there is no obvious increase of expression in normal liver tissue.^18^

### SMMC7721 and BEL7402 cells can be distinguished from LO2 cells by fluorescence when treated with the COX-2 specificity probe

**Fig. 2:**
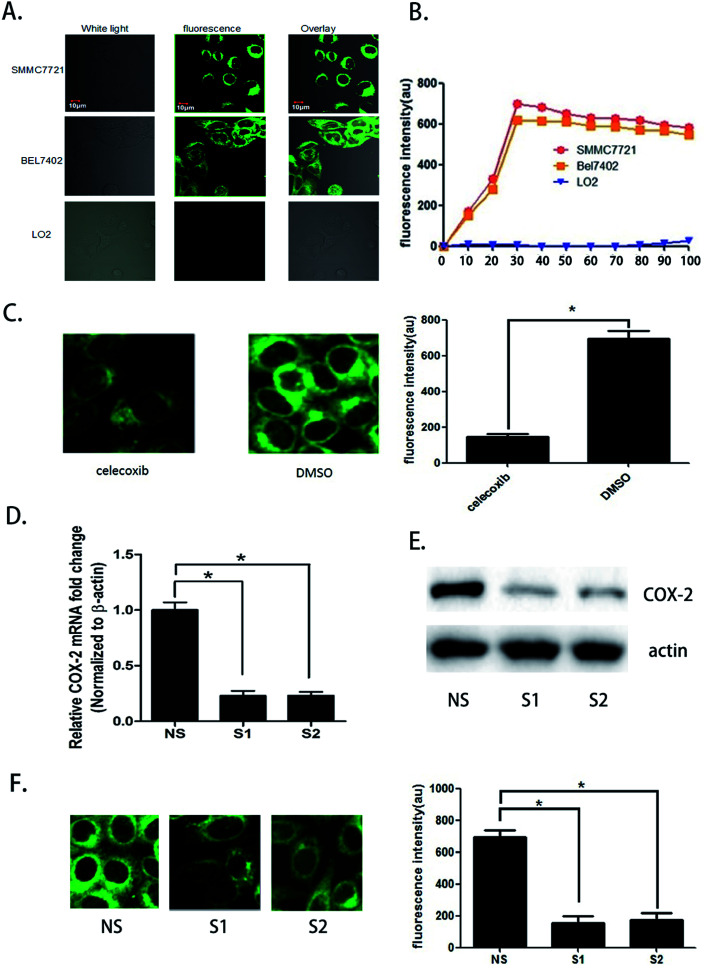
SMMC7721 and BEL7402 cells can be distinguished from LO2 cells by fluorescence when treated with a COX-2 specificity probe. (A) Fluorescent image of SMMC7721, BEL7402, and LO2 cells. COX-2 FP = 5.0 μM; incubation time = 30 min. (B) Time-dependent fluorescence intensity in SMMC7721, BEL7402, and LO2 cells. (C) Fluorescence image and intensity when COX-2 inhibitor (celecoxib, concentration 5.0 μg mL^−1^) was added to SMMC7721 cells. (D) The expression levels of the PTGS2 in SMMC7721 were depressed at the knockdown cells (S1, S2) compared with the control cells (NS). (E) Western blot analysis of expression of COX-2 proteins in NS, S1, and S2. (F) Fluorescent image and intensity in PDGF2 knocking down cells (S1, S2) compared with control cells (NS).

We seeded SMMC7721, BEL7402, and LO2 cells in different fluorescence culture dishes and waited until the cells were in a logarithmic phase. Then, 5.0 μM COX-2 FP was added to each culture dish, and the cells were cultured. Fluorescence microscopy was used to continuously observe the cells (*λ*_ex_ = 480 nm, *λ*_em_ = 550 ± 20 nm). Fluorescence could be observed in the SMMC7721 and BEL7402 cell lines, and the fluorescence intensity enhanced over time. At the 30 minute point when the probe was administered, the fluorescence intensity increased to the highest level. At 90 minutes, the intensity was still more than 90%; however, there was no fluorescence in the LO2 cells during the entire process (Fig. 2A and B).

The difference in fluorescence emission shows that COX-2 FP can distinguish hepatocellular carcinoma cells SMMC7721 and BEL7402 from normal liver cells LO2. To confirm that the phenomenon involved different expression levels of COX-2, we treated the cells with celecoxib, a COX-2 inhibitor (at a concentration of 3 μM) and found that the strength of fluorescence in SMMC7721 cells was reduced by nearly half compared with the control (Fig. 2C). Two siRNAs were also used to knock down the expression of COX-2 in SMMC7721 cells. The results show that when COX-2 expression declined, the strength of the fluorescence emission was also reduced (Fig. 2D–F). The data shown above indicate that human hepatocellular carcinoma cells can be distinguished from normal liver cells using the COX-2-specific fluorescence probe. We hypothesized that this may be a new and efficient method to clinically distinguish hepatocellular carcinoma from normal tissue during surgery.

### COX-2 FP has no distinct cytotoxic effect on normal cells

**Fig. 3:**
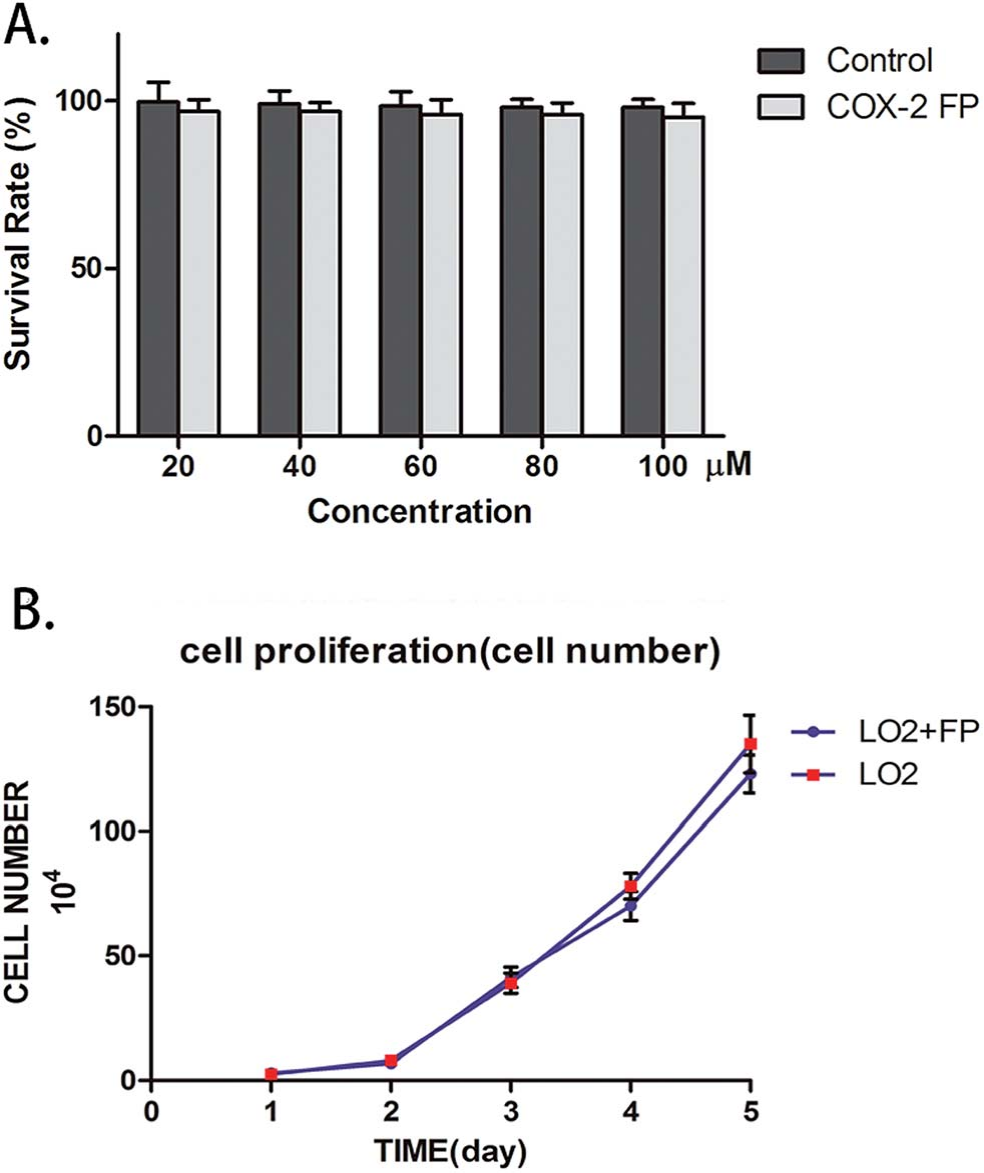
Determining the toxic effect of COX-2 FP on LO2 cells. (A) The survival rate of LO2 cells that had been treated with 20, 40, 60, 80, and 100 μM of COX-2 FP. Co-incubation time = 10 hours. (B) Proliferation of LO2 cells when COX-2 FP was added (concentration = 50 μM); the cell number was counted by the V-cell.

To test if COX-2 FP exerted any toxic effects upon normal cells, we counted the number of LO2 cells to test their survival rate. LO2 cells were combined with varying amounts of COX-2 FP, and the mixture was incubated for approximately 12 hours; the different concentrations of COX-2 FP that were used were 20 μM, 40 μM, 60 μM, 80 μM and 100 μM. The survival rate did not change when comparing the LO2 cells treated with COX-2 FP with the control group (Fig. 3A). To test the long-term effect of the probe, we also monitored the proliferation of the COX-2 FP group (the concentration was 50 μM just as we tested before) and the control group (the same volume of DMSO was added). The five-day proliferation datum also showed no significant difference (Fig. 3B).

### The COX-2-specific fluorescence probe can distinguish heteroplastic hepatocellular carcinoma in a mouse model

**Fig. 4:**
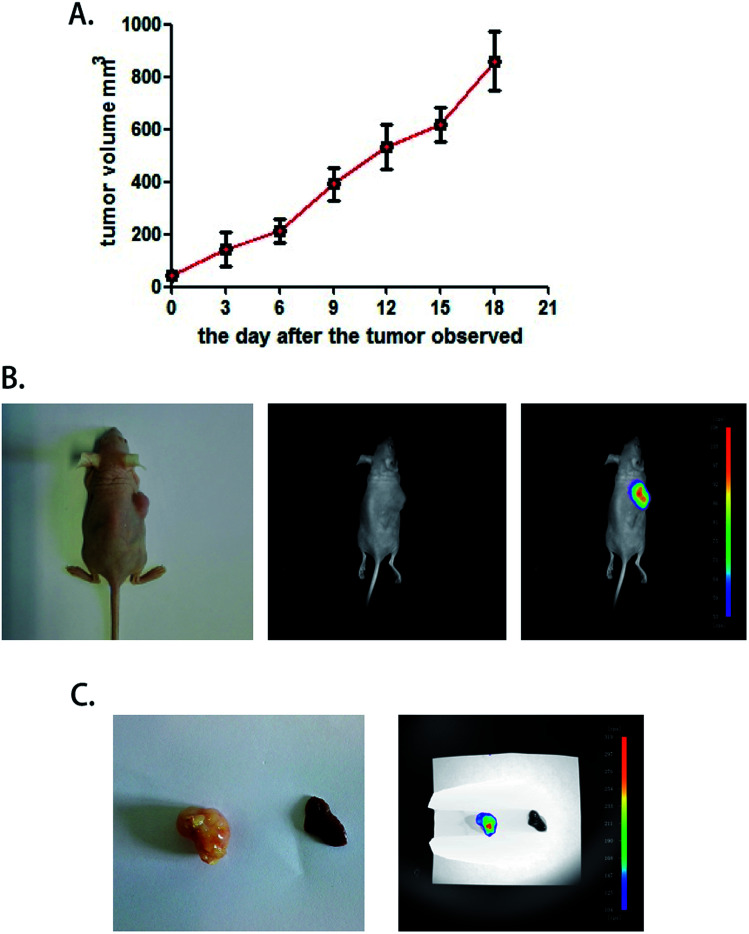
A COX-2-specific fluorescence probe can distinguish heteroplastic hepatocellular carcinoma in the mouse model. (A) The tumor volume curve. The tumor volume was calculated every three days by the formula *V* = 1/2*ab*(*a* + *b*) after small nodules were found at the injection site. The data are presented as the mean tumor volume ± SEM. *n* = 10. (B) The fluorescence image of a xenograft tumor *in vivo*. COX-2 FP (30 μM) was injected intravenously (50 μL). The images were collected 30 minutes after the injection. (C) Fluorescence imaging of a xenograft tumor and liver tissue sample obtained from the COX-2 FP tail injection mouse model.

To test the function of this probe *in vivo*, a heteroplastic carcinoma nude mouse model was built by hypodermic injection of BALB/c mice with the human hepatocellular carcinoma cell line SMMC772. We collected SMMC7721 cells that were in the logarithmic phase and injected 2 × 10^6^ cells into the armpit of the front leg of mice. Seven to nine days after the injection, small tumor nodules could be distinguished, and the tumor formation rate was 100%. The tumor volume was calculated every three days by the formula *V* = 1/2*ab*(*a* + *b*) (Fig. 4A). A fluorescence test was performed when the tumor volume increased to 900 mm^3^, and the mice were still adequately nourished. Next, 30 μM COX-2 FP was administered by tail intravenous injection, and after 30 minutes, the mouse was tested with the Small Animals Living Imaging System (Night OWL IILB983), (*λ*_ex_ = 480 nm, *λ*_em_ 550 ± 20 nm). The tumors could be differentiated by the significant fluorescence emission from COX-2 FP (Fig. 4B). This phenomenon may indicate that COX-2 FP can be activated at the tumor lesions, as it emitted fluorescence at the time point of 30 minutes after intravenous injection *via* the tail.

For testing purposes, the specificity and the capacity of distinguishing tumor lesions from normal liver tissue were examined. A liver sample and a tumor sample obtained with a hypodermic syringe were both collected and directly tested with the imaging system (no liver metastasis were used). The tumor sample emitted intense fluorescence but there was no fluorescence emission at all from the normal liver tissue (Fig. 4C). These results demonstrate that COX-2 FP can cause enrichment of the tumor tissue when injected by tail vein and emits fluorescence that can be used to identify the hepatocellular carcinoma from normal liver tissue in a mouse model, indicating that this method could potentially be used in this way to diagnose tumors *in vivo*.

### Fluorescence emission conditions and imaging depth in human liver tumor samples

**Fig. 5:**
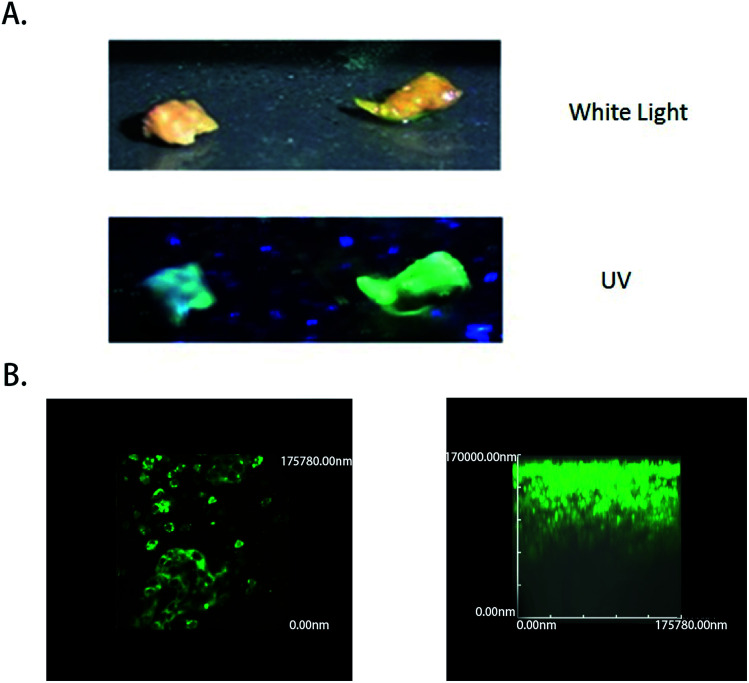
The fluorescence emission conditions and imaging depth in human liver tumor samples. (A) Visualization of human hepatocellular carcinoma resection by the naked eye under ultraviolet illumination. (B) Tumor slices stained with COX-2 FP (30 μM) fluorescence images. Excitation wavelength = 480 nm; scan range = 550 nm.

To test the ability of the COX-2-specific fluorescence probe to clinically distinguish liver tumor cancer specimens from normal liver tissue specimens that were obtained from patients, samples were tested by superficially spraying them with 50 μM COX-2 FP, followed by observation. All the tumor specimens were confirmed to be cancerous by pathological examination, and normal hepatocellular carcinoma specimens were obtained by resection that was performed because of injury. A 350 nm light was provided by a hand-held ultraviolet lamp and was used as the excitation source, resulting in fluorescence when the light was directed to the tissue that had been sprayed with COX-2 FP. A clear difference could be distinguished with the naked eye between the hepatocellular carcinoma sample and normal liver tissue (Fig. 5A). To measure how deep the fluorescence is able to penetrate in this situation, fresh tumor tissue was cut into slices, dipped in 50 μM of COX-2 FP solution, and then observed under a fluorescence microscope (Fig. 5B). The tumor slices could be clearly visualized by green fluorescence at a depth of 100 000 nm. These results demonstrate that COX-2 FP is membrane permeable and suitable for direct fluorescent depth imaging of extremely small amounts of COX-2 in tissues.

## Conclusion

Hepatocellular carcinoma is one of the most common malignant tumors worldwide. Although the standardized therapeutic mode based on surgical resection has been implemented, approximately 60 million people die from hepatocellular carcinoma every year.^19^ The residuals missed during surgery contribute a great part to the poor prognosis. A specific fluorescence probe is composed of a fluorophore and a recognition part. The fluorophore is quenched under normal conditions. When the recognition part connects with a specific target, the structure of the probe changes and the fluorophore emits fluorescence.^20^ This type of probe has been used comprehensively in many research fields, and some studies have shown that specific fluorescence can be used in cancer cell imaging.^11,12,21^ Therefore, we attempted to determine if this type of probe can be used to distinguish hepatocellular carcinoma from normal tissue, especially during surgery. COX-2 is an excluded enzyme that is highly expressed in a tumor but not expressed in most normal tissue. Many studies have demonstrated that COX-2 is highly expressed in hepatocellular carcinoma and plays a crucial role in all stages of liver cancer oncogenesis.^15,22^ Here, we chose a COX-2-specific probe to test if it could distinguish hepatocellular carcinoma both *in vitro* and *in vivo*. We tested the fluorescence emission in hepatocellular carcinoma cell lines, a heterograft mouse model, and specimens obtained from HCC patients after surgical resection. The results show that a hepatocellular carcinoma cell line and heterograft tumors in a mouse model can be distinguished from normal liver tissue by the fluorescence. The difference in fluorescence intensity can be detected by the naked eye when viewing hepatocellular carcinoma samples and normal liver tissues in real time that are illuminated by a hand-held ultraviolet lamp, which activates the dye. With the results shown above and the inherent advantage of the fluorescence test that includes the high sensitivity, high spatial resolution, low cost, portability, simple equipment, rapidity, and absence of ionizing radiation, the COX-2-specific fluorescence probe may be a potential way to distinguish hepatocellular carcinoma especially at the domain of intraoperative assistant imaging.

## Conflicts of interest

The authors declare no conflicts of interest and make no financial disclosures.

## Supplementary Material

RA-008-C7RA07819F-s001
