# Supplementary material for: Fluorescence imaging of hepatocellular carcinoma with a specific probe of COX-2
Source: RSC Adv. 2018 Jan 3;8(2):994–1000. doi: 10.1039/c7ra07819f (PMC9076980; doi:10.1039/c7ra07819f)

Electronic Supplementary Material (ESI) for RSC Advances.
This journal is © The Royal Society of Chemistry 2017

Supplementary Figure 1. The molecular structure of the COX-2 -specific fluorogenic probe.

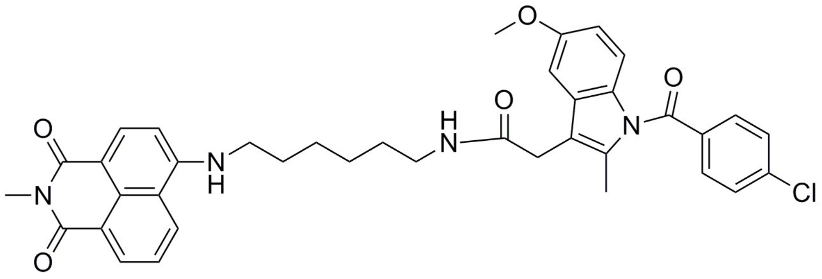

Supplementary Figure 2. Imaging tumors in vivo. COX-2 FP (30 μM) was injected intravenously (30 μL). The incubation time was 30 min.

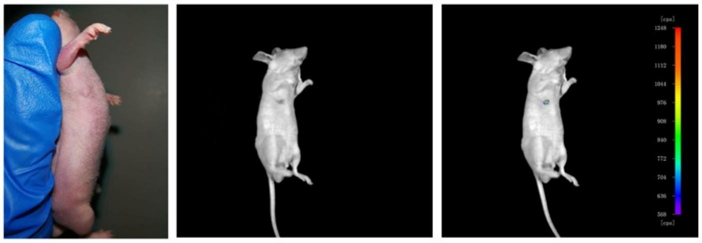

Supplement: RA-008-C7RA07819F-s001 [file RA-008-C7RA07819F-s001.pdf]
